# Supplementary figures and images for: Pilot Study of Metabolomic Clusters as State Markers of Major Depression and Outcomes to CBT Treatment
Source: Front Neurosci. 2019 Sep 12;13:926. doi: 10.3389/fnins.2019.00926 (PMC6751322; doi:10.3389/fnins.2019.00926)

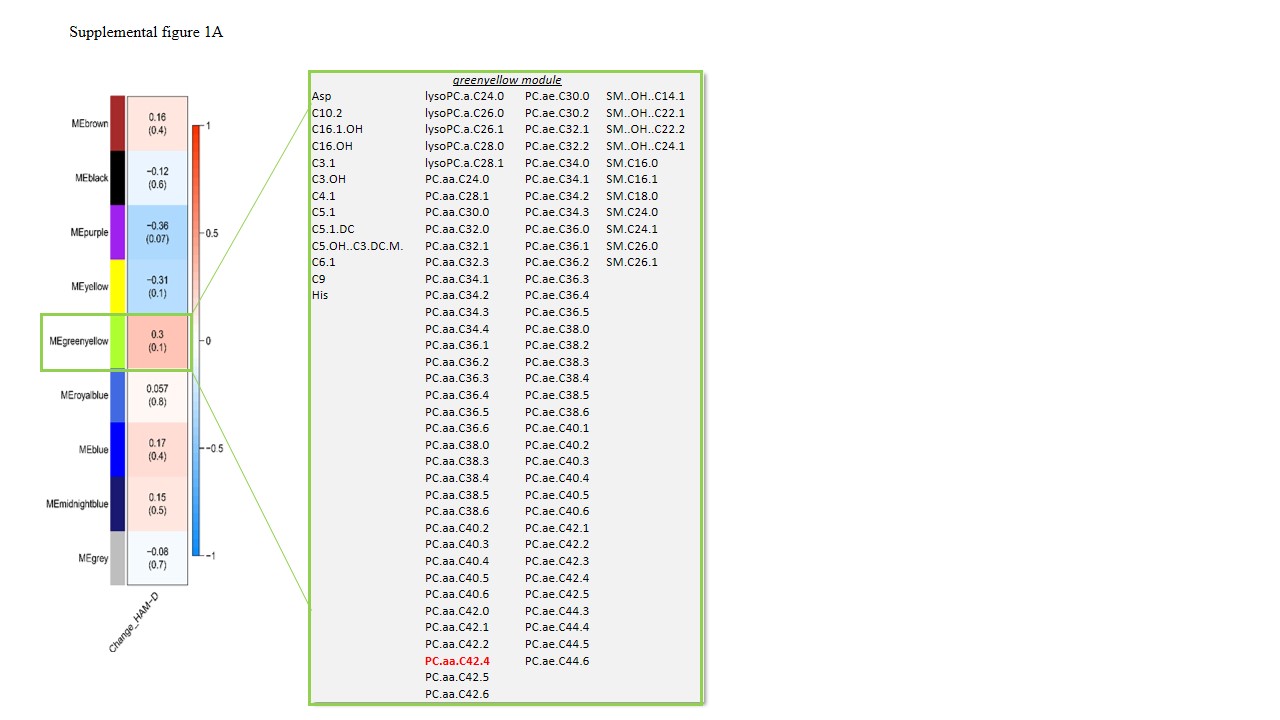

Supplement: FIGURE S1 — (A) A heatmap showing correlations between the metabolite modules and the changes in symptom severity (HAM-D17) scores, highlighting the greenyellow module. The member metabolites of this large module is presented in the adjacent box. (B) A heatmap showing the pairwise Pearson correlations between the member metabolites of the greenyellow module. The empty cells indicate that the p-values of the correlations for that metabolite-pair was not statistically significant (p > 0.1). [file Image_1.JPEG]

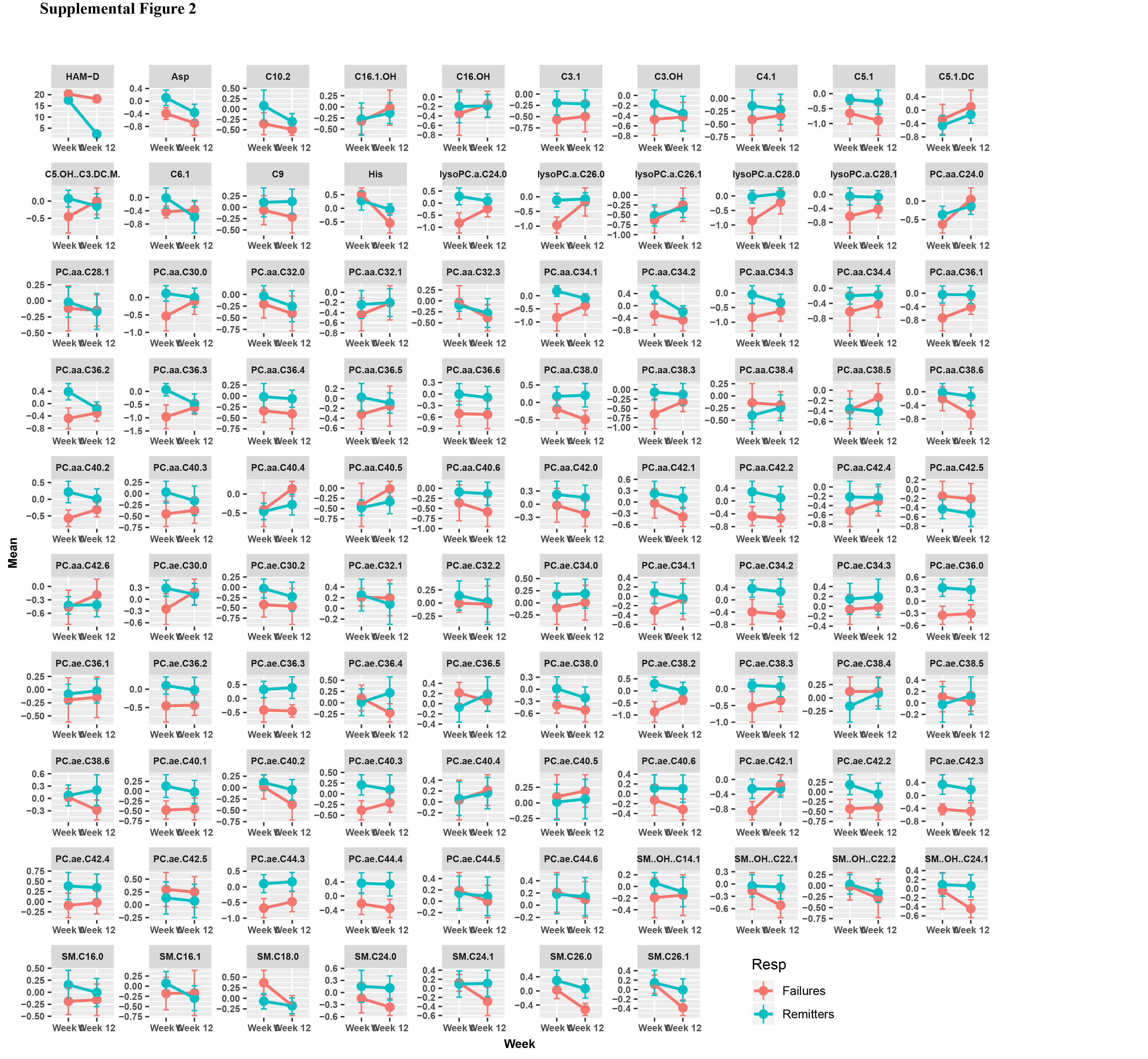

Supplement: FIGURE S2 — The trajectories of each member metabolite of the greenyellow module, consisting of mostly lipids, or lipid-related metabolites, with its mean (± SEM) at baseline and week 12 are presented for remitters and treatment-failures. [file Image_3.JPEG]

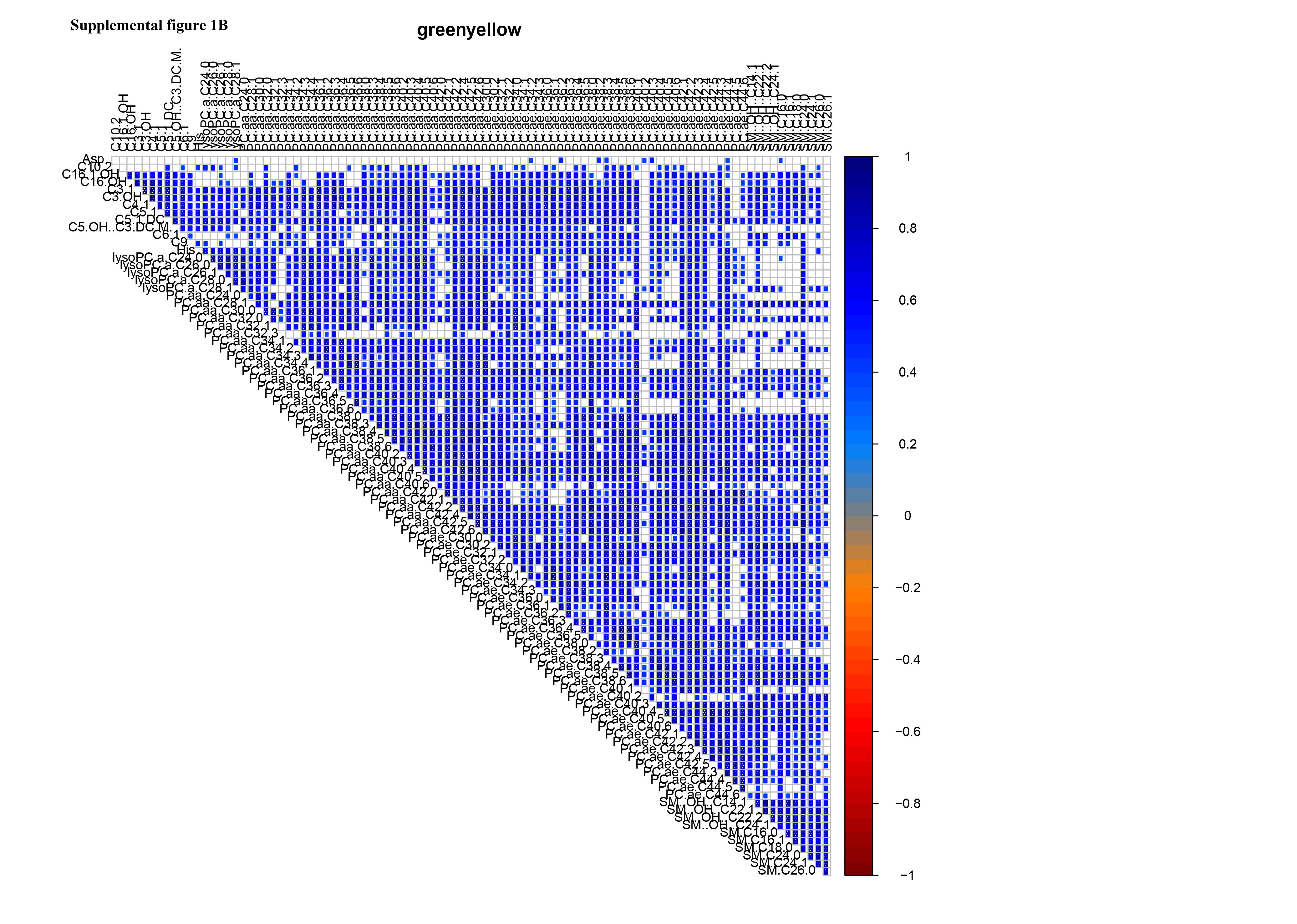

Supplement: Supplementary file 3 [file Image_2.JPEG]
